# Enhancing Empathy and Emotional Awareness in Intrapartum Care Through Role-Reversal Simulation: A Mixed-Methods Study with Midwifery Students

**DOI:** 10.3390/healthcare14172707

**Published:** 2026-08-25

**Authors:** Yeliz Yıldırım Varışoğlu, Selinay Aktaş Demir, İffet Güler Kaya

**Affiliations:** 1 Faculty of Nursing, Istanbul University, Istanbul 34452, Türkiye; yelizy@istanbul.edu.tr; 2 Department of Midwifery, Faculty of Health Sciences, Istanbul Atlas University, Istanbul 34408, Türkiye; 3 Department of Midwifery, Faculty of Health Sciences, Istanbul Medipol University, Istanbul 34083, Türkiye; iffetkaya@medipol.edu.tr

**Keywords:** empathy, emotional awareness, intrapartum care, simulation-based learning, midwifery education, mixed-methods

## Abstract

**Highlights:**

**What are the main findings?**

- Role-reversal simulation significantly improved midwifery students’ emotional availability and responsiveness in intrapartum care.
- Students reported greater emotional engagement, increased awareness of women’s experiences during labour, and enhanced reflection on their professional roles.

**What are the implications of the main findings?**

- Experiential and reflective learning supported the development of woman-centred care practices.
- Role-reversal simulation supported reflective learning and increased awareness of respectful and woman-centred intrapartum care.

**Abstract:**

Background/Objectives: This study evaluated the effects of a role-reversal simulation-based educational intervention on midwifery students’ emotional availability and responsiveness in intrapartum care and explored their experiences of the simulation through qualitative inquiry. Methods: A mixed-methods quasi-experimental one-group pretest–post-test design was used. Forty-nine midwifery students participated in a simulation in which they alternately assumed the roles of a labouring woman and a midwife. Qualitative data were obtained through reflective forms and focus group interviews and analyzed using Braun and Clarke’s six-step thematic analysis. Results: Post-intervention emotional availability and responsiveness scores significantly increased compared with pre-intervention scores (*p* < 0.001), with a large effect size (Cohen’s d = 1.25). Qualitative findings suggested that students perceived greater empathic awareness, a deeper understanding of women’s labour experiences, and increased awareness of respectful and woman-centred care. Participants also described role-reversal simulation and reflective learning as valuable experiences that promoted emotional engagement and greater awareness of their professional roles. Conclusions: Role-reversal simulation may be a useful educational strategy for strengthening emotional availability and responsiveness in intrapartum care among midwifery students. The qualitative findings further suggest that this learning experience may support empathy-related awareness and reflective learning.

## 1. Introduction

Empathy and emotional awareness during labour are critical components of high-quality intrapartum care and play a key role in shaping women’s childbirth experiences. Respectful, humane, and reassuring care during childbirth supports women’s psychological well-being, reduces the risk of postpartum complications, and helps prevent childbirth from being perceived as a traumatic event [1].

Empathy is considered a core professional competency in midwifery practice, directly influencing caring behaviours and forming the foundation of respectful maternity care [2]. Emotional awareness further enhances this competency by enabling midwifery students to recognize women’s physical and emotional needs during labour and provide individualized, holistic care [3]. Qualitative evidence indicates that higher levels of empathy among midwifery students are associated with more positive childbirth experiences and an increased sense of safety for women [4,5]. Recent evidence further supports the educational value of simulation-based learning for empathy development. A recent systematic review and meta-analysis including 28 studies concluded that simulation-based interventions significantly improve empathy among nursing students, particularly when learners actively engage in realistic clinical scenarios and structured reflective activities. These findings reinforce the role of simulation as an evidence-based educational strategy for promoting empathic and patient-centred care [6].

Despite its recognized importance, the development of empathy and emotional awareness remains a challenge in traditional midwifery education, which often prioritizes technical skills over affective competencies. Students may encounter difficulties in understanding the emotional complexity of childbirth and responding appropriately to women’s psychological needs [7]. This gap highlights the need for educational approaches that integrate both cognitive and emotional dimensions of care.

In this context, simulation-based learning has emerged as an effective and innovative educational strategy in midwifery education. Simulation provides a safe and structured environment where students can actively engage in realistic clinical scenarios, develop decision-making skills, and enhance communication and emotional competencies. Actor-based simulations in emotionally challenging situations, such as perinatal loss, have been shown to reduce feelings of helplessness and strengthen professional responses [8,9]. Similarly, pregnancy simulators have been reported to increase empathy and foster more positive attitudes toward pregnant women [10,11], while cultural empathy programmes contribute to improving the quality of care provided to women from diverse backgrounds [12].

Although existing studies support the effectiveness of simulation-based approaches, most simulation research in midwifery education has focused on communication skills, clinical competence, cultural empathy, or perinatal loss scenarios. Evidence regarding role-reversal simulations specifically designed for intrapartum care remains scarce, and little is known about their influence on empathy and emotional awareness. Furthermore, the mechanisms through which simulation enhances empathy, such as perspective-taking, emotional engagement, and reflective learning, have not been sufficiently explored [13].

Role-reversal simulation, in which students experience both the labouring woman and the midwife perspectives, may offer a unique opportunity to address this gap. Experiencing labour from the perspective of a pregnant woman may allow students to internalize feelings of pain, vulnerability, and uncertainty, while the midwife role enables them to integrate emotional awareness into clinical decision-making and caregiving behaviours. This bidirectional experiential process may contribute to the development of empathy, ethical sensitivity, and woman-centred care practices. Beyond technical skill acquisition, simulation-based learning facilitates emotional processing, communication, perspective-taking, and reflective learning by allowing students to experience emotionally demanding clinical situations within psychologically safe environments. Such experiences promote deeper engagement with patients’ perspectives and contribute to the development of emotionally competent healthcare professionals [14].

Therefore, strengthening empathy and emotional awareness through experiential and simulation-based learning should be considered a fundamental objective in midwifery education. This study aimed to evaluate the effects of a role-reversal simulation-based educational intervention on emotional availability and responsiveness in intrapartum care among midwifery students. In addition, qualitative methods were used to explore students’ perceptions and experiences of the simulation process.

## 2. Materials and Methods

### 2.1. Design

This study employed a sequential explanatory mixed-methods design according to Creswell and Plano Clark. The quantitative phase consisted of a one-group pretest–post-test quasi-experimental study evaluating changes in emotional availability and responsiveness following the simulation intervention. Subsequently, qualitative data were collected through reflective forms and focus group interviews to explain and enrich the quantitative findings by exploring students’ experiences and perceptions of the simulation. Integration of both datasets occurred during the interpretation stage.

### 2.2. Setting and Participants

The study was conducted at the Department of Midwifery, Faculty of Health Sciences, XXX University, Türkiye, between January and April 2025. The study population consisted of 238 midwifery students enrolled in the department. Because the intervention was integrated into the practical component of the curriculum, no formal a priori sample size calculation was performed. Instead, all eligible students who met the inclusion criteria and voluntarily agreed to participate during the study period were recruited using convenience sampling. Consequently, the final sample consisted of 49 students. Eligible students were informed about the study during scheduled class sessions and provided written informed consent before participation.

Inclusion criteria: Students who had successfully completed the theoretical courses and were willing to participate were considered eligible.

- Voluntary participation.
- Completion of the following courses:
- Midwifery Care in Labor and Postpartum.
- Midwifery Care in High-Risk Labor and Postpartum.

Exclusion criteria:

- Refusal to participate.
- Not completing the required courses.

All participants were informed about the study and provided written and verbal informed consent prior to participation.

### 2.3. Simulation-Based Educational Intervention

The intervention was conducted in the midwifery simulation laboratory and was designed as a role-reversal simulation experience. The simulation sessions were conducted by two faculty members experienced in simulation-based midwifery education. To ensure consistency across groups, all sessions followed the same standardized scenario, prebriefing procedure, simulation protocol, and structured debriefing process. Each session consisted of a 15 min prebriefing, a 30 min role-reversal simulation, and a 20 min structured debriefing. The post-intervention EAR-IC questionnaire was administered immediately after completion of the debriefing session.

#### 2.3.1. Prebriefing

Before the simulation, students received a structured orientation session that included:

- Learning objectives;
- Scenario overview;
- Roles and expectations;
- Psychological safety and confidentiality principles.

#### 2.3.2. Role-Reversal Simulation Process

The intervention consisted of two sequential simulation roles:

Pregnant Woman Role

- Students experienced a simulated labour process using the MamaBirthie Birth Simulator (Laerdal Medical, Stavanger, Norway) and an empathy model. This phase allowed students to:
- Experience physical discomfort and positioning during labour;
- Develop awareness of vulnerability and emotional needs;
- Reflect on privacy, dignity, and communication.

Midwife Role

- Students then assumed the role of the midwife and managed the childbirth process. This phase focused on:
- Clinical decision-making;
- Emotional support and communication;
- Respectful and woman-centred care practices.

Each student completed both roles, enabling perspective-taking and experiential learning.

#### 2.3.3. Debriefing Process

The debriefing session followed a structured reflective approach encouraging students to discuss their emotional reactions, communication experiences, and learning outcomes while linking the simulation experience with future clinical practice.

This process aimed to facilitate reflective learning, emotional processing, and the integration of newly acquired knowledge.

### 2.4. Data Collection

**Figure 1:**
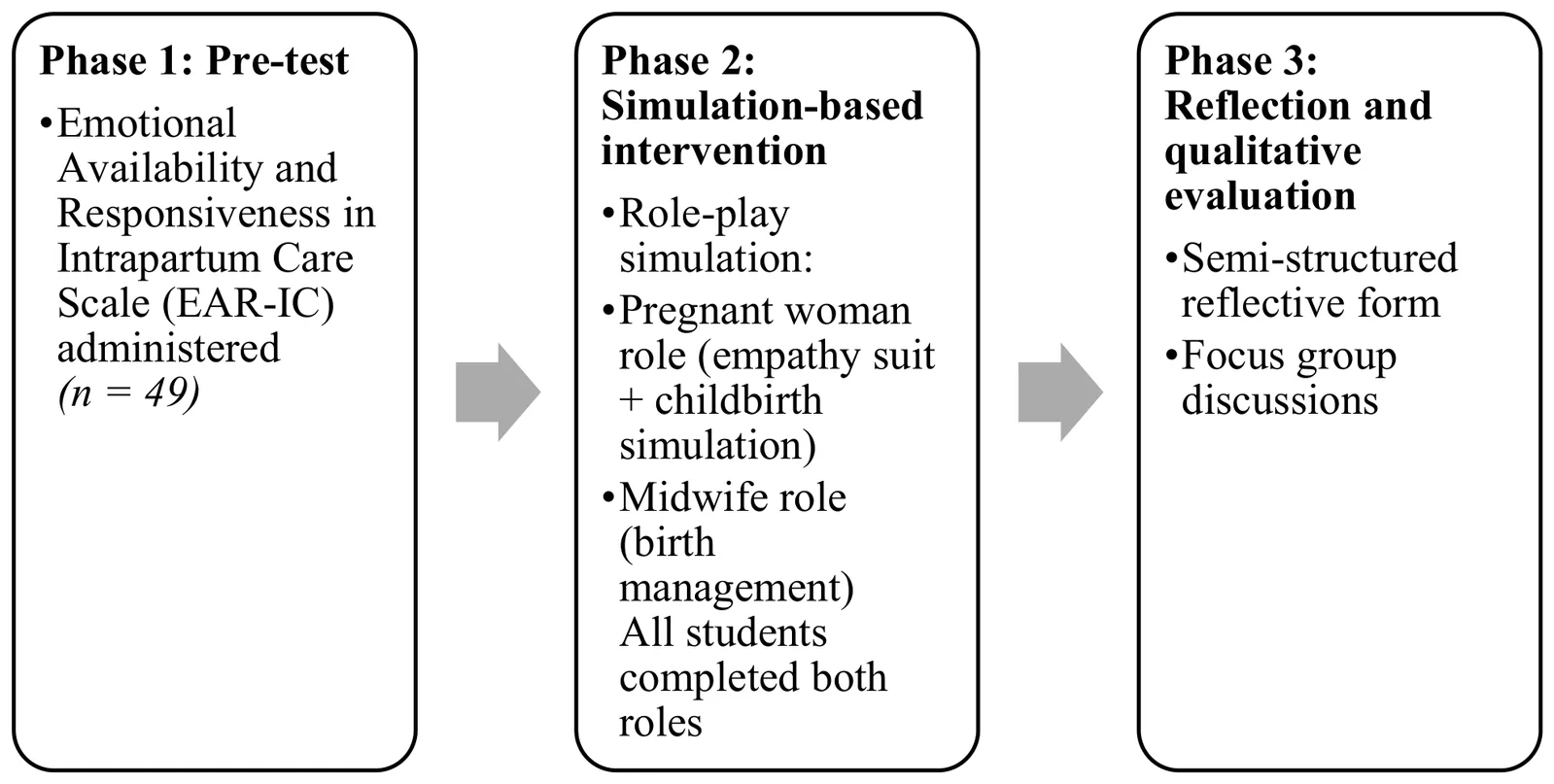
Flow diagram of the simulation-based educational intervention.

Data were collected in three phases:

The integration of quantitative and qualitative findings enabled a comprehensive evaluation of the educational intervention (Figure 1).

These methods aimed to explore students’ emotions, perceptions, and learning experiences. Qualitative data were collected through seven focus group interviews, each comprising seven participants. Each interview lasted approximately 60 min. Data collection continued until thematic saturation was achieved, with no new codes or themes emerging during the final focus group discussions.

### 2.5. Data Collection Instruments

#### 2.5.1. Emotional Availability and Responsiveness in Intrapartum Care Scale (EAR-IC)

This is an eight-item, single-factor Likert-type scale adapted into Turkish by Yıldırım Varışoğlu and Vural (2024) [15]. Scores range from 8 to 40, with higher scores indicating greater emotional responsiveness [15]. In the present study, the internal consistency of the scale was re-evaluated, yielding a Cronbach’s alpha coefficient of 0.82, indicating good internal consistency.

#### 2.5.2. Semi-Structured Interview Form

The interview guide was developed by the research team following a review of the literature on empathy, simulation-based learning, and intrapartum care. Questions were pilot-tested with two students to assess clarity before data collection.

- A researcher-developed form consisting of open-ended questions exploring:
- Emotional experiences during simulation;
- Perceptions of intrapartum care;
- Awareness of empathy and communication;
- Reflections on professional roles.

### 2.6. Data Analysis

#### 2.6.1. Quantitative Analysis

Quantitative data were analyzed using IBM SPSS Statistics version 30.0 (IBM Corp., Armonk, NY, USA). Descriptive statistics (mean, standard deviation, minimum, and maximum values) were calculated to summarize participant characteristics and study variables. The distribution of the data and the normality of the pre–post difference scores were assessed using the Kolmogorov–Smirnov and Shapiro–Wilk tests. The assumptions for the paired-samples *t*-test were examined prior to analysis. Based on these assessments, appropriate statistical tests were selected. For normally distributed variables, paired-samples *t*-tests were performed to compare pre- and post-intervention scores, whereas the Wilcoxon signed-rank test was used for variables that did not meet normality assumptions. Statistical significance was set at *p* < 0.05. Cohen’s d was calculated to estimate the magnitude of the intervention effect. The dataset was examined for completeness before analysis. No missing data were identified; therefore, all participants were included in the final analyses.

#### 2.6.2. Qualitative Analysis

Qualitative data were analyzed using Braun and Clarke’s six-step thematic analysis approach with MAXQDA 2024 software (VERBI Software GmbH, Berlin, Germany). Initially, all interviews were transcribed verbatim and repeatedly read to achieve familiarity with the data. Initial codes were generated independently by two researchers using MAXQDA 2024 software. Codes with similar meanings were grouped into categories and subsequently organized into overarching themes. Any disagreements were resolved through discussion until consensus was reached. To enhance the trustworthiness of the findings, investigator triangulation, participant validation, and the use of representative quotations were employed.

#### 2.6.3. Integration of Data

Finally, quantitative and qualitative findings were integrated during the interpretation phase to provide a comprehensive understanding of the impact of the simulation-based educational intervention. The quantitative and qualitative findings were integrated during interpretation to compare convergence and complementarity between the two datasets. The qualitative findings were primarily used to explain and contextualize the quantitative results rather than to provide objective evidence of additional outcomes.

### 2.7. Ethical Considerations

Ethical approval was obtained from the Non-Interventional Clinical Research Ethics Committee (Approval No: 2025/58766). Institutional permission was granted by the Faculty of Health Sciences. Participants provided written and verbal informed consent. Confidentiality and anonymity were ensured throughout the study.

## 3. Results

### 3.1. Quantitative Findings

The results demonstrated a significant improvement in students’ emotional availability and responsiveness following the simulation-based intervention. The mean pre-test score on the Emotional Availability and Responsiveness in Intrapartum Care Scale was 30.16 ± 3.39, whereas the mean post-test score increased to 36.35 ± 3.71.

As shown in Table 1, paired-samples *t*-test analysis revealed a statistically significant difference between pre- and post-intervention scores (t (48) = −8.77, *p* < 0.001). The mean difference between the two measurements was 6.18 (95% CI: 4.77 to 7.60), indicating a substantial increase in emotional responsiveness after the intervention. Effect size analysis indicated a very large magnitude of change (Cohen’s d = 1.25), indicating a substantial increase in students’ emotional availability and responsiveness following the simulation-based educational intervention.

### 3.2. Qualitative Findings

The qualitative analysis yielded five overarching themes and associated subthemes that reflect students’ multidimensional learning experiences during the simulation-based intervention. As presented in Table 2, these themes encompass both experiential and professional dimensions of intrapartum care learning.

#### 3.2.1. Theme 1: Birth Environment Experiences

Students emphasized that both the physical and emotional characteristics of the birth environment significantly shaped their experiences. Calm, quiet, and dimly lit settings were associated with feelings of safety and comfort, whereas bright lighting, noise, and crowded environments increased anxiety and discomfort. Privacy was consistently highlighted as a central component of emotional security; its absence intensified feelings of vulnerability, shame, and loss of control. In addition, empathic communication and a supportive presence were perceived as essential for a positive birth experience.

“A dim and quiet environment eased the burden of labor; I felt safer.” (P12)

“Giving birth under a crowd and inappropriate gazes increased my shame and anxiety.” (P28)

“When someone spoke calmly and stayed with me, I felt less afraid.” (P7)

#### 3.2.2. Theme 2a: Conducting Birth (Midwife Role)

While assuming the midwife role, students reported a strong sense of responsibility for the safety of both mother and baby. Initial feelings of anxiety, fear of making mistakes, and uncertainty gradually evolved into increased confidence and professional awareness. Students also recognized the importance of providing emotional support, maintaining calm communication, and being present for the woman during labour. Some participants described the experience as meaningful and, at times, carrying a spiritual dimension.

“The health of the mother and baby was entirely my responsibility; that awareness brought both anxiety and strength.” (P1)

“At first I was very nervous, but as I continued, I felt more confident and in control.” (P9)

“Being there for the woman emotionally was as important as the clinical interventions.” (P15)

#### 3.2.3. Theme 2b: Professional Development

The simulation contributed significantly to students’ professional development. Participants reported a clearer understanding of their professional roles and responsibilities and described feeling more confident when reflecting on clinical situations. Some students perceived that the simulation encouraged them to think more carefully about clinical decision-making; however, objective clinical competence or decision-making performance was not evaluated in this study. The experience helped them differentiate between supportive and non-supportive practices and increased their awareness of ethical responsibilities in intrapartum care.

“I understood better what it means to take responsibility as a midwife.” (P5)

“This experience helped me see what good care really looks like.” (P18)

#### 3.2.4. Theme 3: Experiencing Birth (Pregnant Woman Role)

Students described the pregnant woman role as one of the most impactful components of the simulation. They reported experiencing intense physical discomfort alongside emotional responses such as fear, anxiety, vulnerability, and uncertainty. This embodied experience enabled them to better understand the emotional and physical needs of women during labour and was frequently identified as a key factor in enhancing empathy and emotional awareness.

“I felt helpless and vulnerable; it made me realize how important support is.” (P21)

“Experiencing the pain changed how I see women in labour.” (P30)

“For my professional life, it was very important to approach the pregnant woman by looking from her perspective.” (P24)

#### 3.2.5. Theme 4: Influencing Factors

Students identified several factors influencing their behaviours, thoughts, and clinical decision-making processes, including knowledge, prior clinical experiences, personal values, empathy, and bias. While theoretical knowledge increased confidence, previous negative observations in clinical settings motivated students to adopt more respectful and supportive approaches. Personal values and empathy were also reported as key determinants guiding professional behaviour.

“What I learned in theory gave me confidence, but this experience made it real.” (P11)

“Seeing negative practices before made me want to do things differently.” (P26)

#### 3.2.6. Theme 5: Observations on Birth Practices

Students critically evaluated observed clinical practices and distinguished between positive and negative approaches. Practices such as lack of privacy, unnecessary interventions, and poor communication were frequently described by participants as harmful and ethically problematic. In contrast, respectful communication, supportive presence, and non-pharmacological pain management were identified as essential components of high-quality intrapartum care.

“Any intervention performed without privacy felt unethical to me.” (P29)

“Respectful communication makes a huge difference in how the woman feels.” (P14)

“Simple support and calmness can change the whole birth experience.” (P6)

### 3.3. Integration of Quantitative and Qualitative Findings

The qualitative findings supported and enriched the quantitative results by providing deeper insight into the mechanisms underlying the observed changes. While statistical analyses demonstrated a significant increase in emotional responsiveness, qualitative data revealed that this improvement was associated with experiential learning, emotional engagement, and perspective-taking facilitated by the role-reversal simulation.

The quantitative findings demonstrated a significant improvement in students’ emotional availability and responsiveness following the intervention. The qualitative findings complemented these results by showing that participants perceived greater awareness of women’s childbirth experiences, reflected more deeply on respectful care, and described increased awareness of their professional roles. These qualitative findings provide contextual understanding of the quantitative results rather than objective evidence of improvements in clinical competence, professional identity, or ethical sensitivity. The integration of quantitative and qualitative data highlights the value of simulation-based experiential learning in fostering both cognitive and affective competencies in midwifery education and supporting the development of compassionate, woman-centred care.

## 4. Discussion

This study demonstrated that a role-reversal simulation-based educational intervention was associated with increased emotional availability and responsiveness among midwifery students, while qualitative findings suggested greater empathy-related awareness and reflective learning. While quantitative findings revealed a statistically significant and substantial increase in emotional availability and responsiveness, qualitative findings provided deeper insight into the experiential mechanisms underlying this improvement.

The observed large effect size suggests that the simulation-based intervention was associated with substantial improvements in emotional availability and responsiveness. These findings support the view that simulation-based education is not only a means of developing technical skills but also an educational approach that promotes emotional engagement and reflective learning.

In particular, the role-reversal structure of the intervention, in which students experienced both the labouring woman and the midwife perspectives, may have played an important role in facilitating perspective-taking and reflective understanding of women’s childbirth experiences, as described by participants.

Consistent with previous studies, empathy-focused educational interventions have been shown to improve empathy levels among midwifery students. Tafazoli et al. (2018) reported increased empathy scores following training, although the magnitude of change was limited [16]. Similarly, Hogan et al. (2018) demonstrated that cultural empathy programmes enhanced empathy, although the effects tended to diminish over time [12]. In contrast, the strong and statistically significant improvement observed in the present study suggests that experiential and immersive learning approaches, such as role-reversal simulation, may produce a more robust and potentially sustained impact. The present findings are consistent with recent evidence demonstrating that simulation-based educational interventions improve empathy by combining experiential learning with guided reflection. A recent systematic review and meta-analysis reported that simulation-based interventions significantly enhance empathy among nursing students, particularly when realistic clinical scenarios are combined with structured reflection. Likewise, recent evidence suggests that simulation promotes emotional processing, communication skills, and perspective-taking, thereby supporting patient-centred care and emotionally competent practice [6,14].

The use of simulation tools, including the empathy model and childbirth simulator, aligns with innovative approaches reported in the literature. Andersen et al. (2019) emphasized that wearable simulation technologies enhance realism and student engagement [17]. In the present study, students’ experiences of physical discomfort, emotional vulnerability, and uncertainty while assuming the pregnant woman role appear to have facilitated a deeper internalization of the childbirth experience. This embodied learning process likely contributed to the observed increase in empathy and emotional awareness.

Students’ transition from initial anxiety and uncertainty to increased confidence and professional awareness is also consistent with the previous findings. Vermeulen et al. (2017) reported that emotional tension experienced during simulation can positively influence learning outcomes by promoting engagement and reflection [18]. Similarly, in the present study, students’ emotional responses during simulation were not barriers but rather facilitators of learning, supporting students’ reflective learning and awareness of emotional aspects of intrapartum care. The educational benefits observed in the present study may also be explained by the structured debriefing process. According to the Healthcare Simulation Standards of Best Practice™, debriefing represents the core learning component of simulation-based education, providing learners with opportunities to process emotional experiences, engage in guided reflection, receive constructive feedback, and integrate experiential learning into future clinical practice. Consequently, the improvements observed in emotional availability and responsiveness may have resulted not only from the role-reversal experience itself but also from the structured reflective discussions conducted after the simulation [19].

Furthermore, findings from Aktaş and Pasinlioğlu (2021) indicate that empathy training among midwives improves empathic communication skills and enhances maternal satisfaction [20]. These findings support the broader clinical relevance of empathy-focused education, suggesting that empathy-focused educational interventions may contribute to more supportive maternity care. However, the present study did not evaluate clinical practice outcomes or maternal experiences. In the present study, students’ increased awareness of respectful communication, privacy, and emotional support further reinforces the importance of integrating such competencies into midwifery education.

The qualitative findings highlighted the importance of environmental, interpersonal, and ethical factors in shaping childbirth experiences. Students demonstrated increased awareness of privacy, communication, and respectful care, while critically evaluating interventionist and non-evidence-based practices. These findings are consistent with Cuerva et al. (2018), who emphasized that simulation enables the teaching of complex concepts such as dignity, respect, and patient-centred care [21]. Similarly, Kumar et al. (2016) highlighted the role of simulation in developing communication and teamwork skills, which were also reflected in students’ experiences in the present study [22].

The findings can also be interpreted within the framework of transformative learning theory. Swordy et al. (2020) suggested that experiential and narrative-based interventions facilitate reflection, empathy development, and awareness of care practices [23]. In the present study, the combination of role-reversal simulation and reflective activities appears to have enabled students to move beyond theoretical understanding and internalize women’s childbirth experiences, thereby encouraging students to reflect on their professional roles and responsibilities. Empathy should also be considered alongside emotional intelligence and emotional regulation. Recent evidence indicates that simulation-based learning not only enhances emotional competencies but also strengthens resilience, self-efficacy, and emotional intelligence while contributing to reduced burnout among healthcare students. These findings suggest that simulation-based educational interventions should aim to promote balanced emotional engagement rather than empathy alone, thereby preparing students for emotionally demanding clinical environments [24].

Recent evidence from Türkiye further supports the importance of strengthening empathy in midwifery education. Bayrakçı Eroğlu and Akbaş (2026) reported moderate levels of empathy among midwifery students and emphasized the need for experiential learning approaches [25]. The findings of the present study provide further support for the effectiveness of simulation-based educational strategies in addressing this gap.

Collectively, the present findings together with recent evidence support role-reversal simulation as an effective experiential learning strategy for strengthening emotional availability, empathy, reflective practice, communication, and broader emotional competencies required for respectful woman-centred intrapartum care. Integrating realistic simulation scenarios with structured debriefing may therefore represent a valuable approach for preparing future midwives to provide compassionate and emotionally responsive maternity care [6,14,19,24].

The integration of experiential learning and reflective processes appears to be particularly effective in transforming empathy from a theoretical concept into an internalized professional competency.

### 4.1. Limitations and Recommendations

This study has several limitations. First, the absence of a control group limits the ability to draw causal inferences regarding the observed changes. Second, the study was conducted in a single institution with a relatively small sample size, which may restrict the generalizability of the findings. In addition, the long-term sustainability of the improvements and their transfer to real clinical practice were not assessed.

Future research should include multicentre designs with larger samples and controlled experimental approaches to strengthen causal interpretations. Longitudinal studies are also recommended to evaluate the persistence of empathy-related gains over time and their impact on clinical performance in real-world settings. In addition, qualitative findings reflected participants’ subjective experiences and should not be interpreted as objective evidence of improvements in clinical competence, professional identity, ethical sensitivity, or decision-making skills. Furthermore, the use of self-report measures may have introduced social desirability bias.

### 4.2. Implications for Simulation Practice

The findings of this study highlight the importance of integrating simulation-based and experiential learning approaches into midwifery education to enhance empathy and emotional awareness in intrapartum care. Role-reversal simulation, in which students experience both the labouring woman and midwife perspectives, appears to be an effective strategy for fostering perspective-taking, emotional engagement, and reflective learning.

Incorporating structured simulation scenarios and guided debriefing into educational programmes may support the development of not only technical skills but also affective and ethical competencies essential for woman-centred care. Simulation-based interventions may therefore contribute to improving the quality of intrapartum care by preparing future midwives to provide compassionate, respectful, and holistic support to women during childbirth. Collectively, recent evidence and the present findings support the integration of simulation-based educational approaches into undergraduate midwifery curricula, not only to improve emotional responsiveness but also to strengthen reflective practice, communication, and broader emotional competencies required for respectful woman-centred care.

## 5. Conclusions

The findings indicate that role-reversal simulation was associated with increased emotional availability and responsiveness among midwifery students. Qualitative findings further suggest that students perceived greater awareness of women’s experiences during labour and engaged in deeper reflection regarding respectful maternity care and their professional roles. Because these latter findings were derived from participants’ subjective experiences rather than objective performance assessments, they should be interpreted cautiously. Future controlled studies incorporating objective measures of clinical competence are needed to further evaluate the educational impact of role-reversal simulation.

## Figures and Tables

**Table 1:** Comparison of students’ pre-test and post-test EAR-IC scores.

Measurement	*n*	Mean ± SD	Mean Difference	t	df	*p*	Effect Size (Cohen’s d)
Pre-test	49	30.16 ± 3.39	6.18			<0.001	
Post-test	49	36.35 ± 3.71		8.77	48		1.25

Note. Values are expressed as mean ± standard deviation. Differences between pre- and post-intervention scores were analyzed using the paired-samples *t*-test. A *p* value of <0.05 was considered statistically significant. Effect size was calculated using Cohen’s d, with values interpreted as small (0.2), medium (0.5), and large (0.8).

**Table 2:** Themes, subthemes, and representative quotations.

Main Theme	Subthemes	Representative Quotations
Theme 1: Birth environment experiences	Physical environment; privacy and safety; emotional atmosphere and communication	“A dim and quiet environment eased the burden of labor; I felt safer.” (P12)
Theme 2a: Conducting birth (midwife role)	Responsibility and anxiety; confidence and professional awareness; empathy and support; spiritual meaning	“The health of the mother and baby was entirely my responsibility; that awareness brought both anxiety and strength.” (P1)
Theme 2b: Professional development	Professional role and responsibility; clinical experience	“This experience helped me understand what being a midwife truly means.” (P18)
Theme 3: Experiencing birth (pregnant woman role)	Physical pain; emotional vulnerability; spiritual meaning; emotional awareness and empathy	“Experiencing the pain changed how I see women in labour.” (P30)
Theme 4: Influencing factors	Knowledge; experience; values; empathy; bias	“What I learned in theory gave me confidence, but this experience made it real.” (P11)
Theme 5: Observations on birth practices	Causes; impacts; improvements; examples; ethics	“Any intervention performed without privacy felt unethical to me.” (P29)

Note. Representative quotations are selected to illustrate key aspects of each theme derived from thematic analysis.

## Data Availability

The data presented in this study are available on request from the corresponding author due to privacy and ethical restrictions related to sensitive participant data.

## Abbreviations

The following abbreviations are used in this manuscript:

CI	Confidence Interval
df	Degrees of Freedom
EAR-IC	Emotional Availability and Responsiveness in Intrapartum Care Scale
MAXQDA	Max Qualitative Data Analysis Software
SD	Standard Deviation
SPSS	Statistical Package for the Social Sciences
